# Structural and Functional Characterization of the Secondary Mutation N126K Selected by Various HIV-1 Fusion Inhibitors

**DOI:** 10.3390/v12030326

**Published:** 2020-03-18

**Authors:** Danwei Yu, Yang Su, Xiaohui Ding, Yuanmei Zhu, Bo Qin, Huihui Chong, Sheng Cui, Yuxian He

**Affiliations:** 1 NHC Key Laboratory of Systems Biology of Pathogens, Institute of Pathogen Biology and Center for AIDS Research, Chinese Academy of Medical Sciences and Peking Union Medical College, Beijing 100730, China; 2 Department of Lab Medicine, Institute of Hematology, Chinese Academy of Medical Sciences and Peking Union Medical College, Tianjin 300020, China

**Keywords:** HIV-1, gp41, fusion inhibitor, resistance, secondary mutation

## Abstract

Peptides derived from the C-terminal heptad repeat (CHR) region of HIV-1 gp41 is potent viral membrane fusion inhibitors, such as the first clinically approved peptide drug T20 and a group of newly-designed peptides. The resistance profiles of various HIV-1 fusion inhibitors were previously characterized, and the secondary mutation N126K in the gp41 CHR was routinely identified during the in vitro and in vivo selections. In this study, the functional and structural relevance of the N126K mutation has been characterized from multiple angles. First, we show that a single N126K mutation across several HIV-1 isolates conferred mild to moderate cross-resistances. Second, the N126K mutation exerted different effects on Env-mediated HIV-1 entry and cell-cell fusion. Third, the N126K mutation did not interfere with the expression and processing of viral Env glycoproteins, but it disrupted the Asn126-based glycosylation site in gp41. Fourth, the N126K mutation was verified to enhance the thermal stability of 6-HB conformation. Fifth, we determined the crystal structure of a 6-HB bearing the N126K mutation, which revealed the interhelical and intrahelical interactions underlying the increased thermostability. Therefore, our data provide new information to understand the mechanism of HIV-1 gp41-mediated cell fusion and its resistance mode to viral fusion inhibitors.

## 1. Introduction

**Figure 1:**
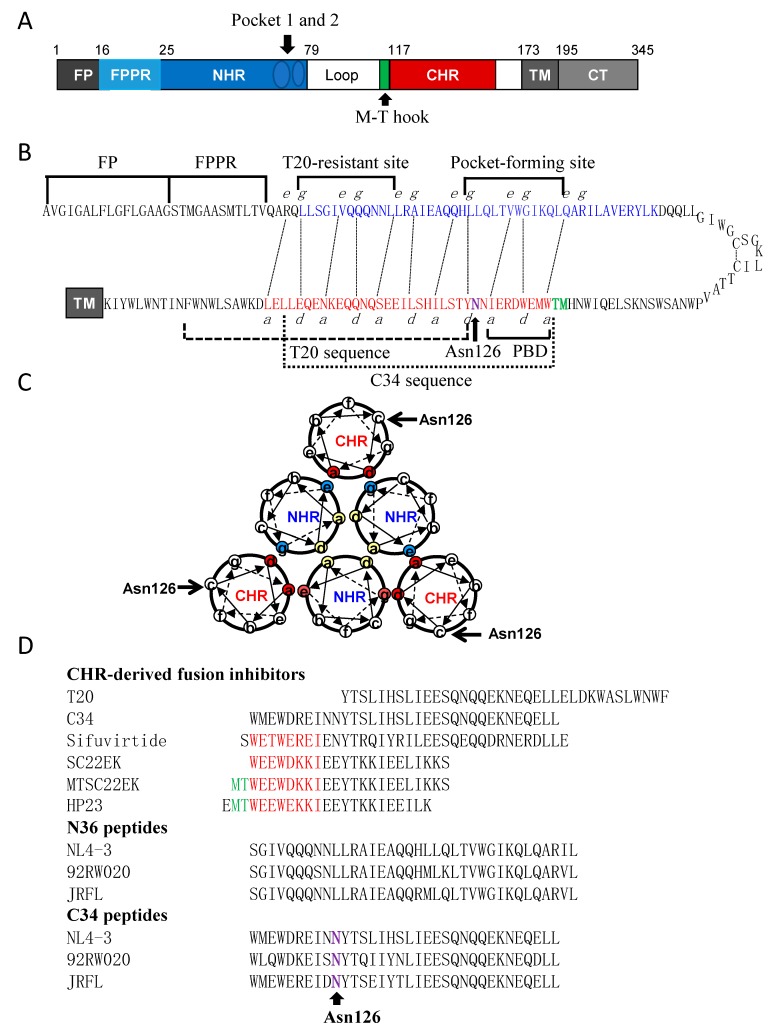
Schematic diagram of HIV-1 gp41 structure and its peptide derivatives. (**A**) Functional domains of gp41 in the numbering of HIV-1_HXB2_ gp41. FP, fusion peptide; FPPR, fusion peptide proximal region; NHR, N-terminal heptad repeat; CHR, C-terminal heptad repeat; TM, transmembrane domain; CT, cytoplasmic tail. Two NHR pocket sites and the M-T hook site are specifically marked. (**B**) A hairpin model of gp41 illustrating the interacting residues between the NHR and CHR sequences. The dashed black lines indicate the interactions between the residues at the “*e*” and “*g*” positions in the NHR (marked in blue) and the “*a*” and “*d*” positions in the CHR (marked in red), respectively. The sequences for FP, FPPR, T20-resistant site, deep pocket-forming site in the NHR and PBD, T20, C34 and Asn126 in the CHR are respectively marked for clarity. (**C**) A helical wheel model of gp41 illustrating the interactions between the NHR and CHR helices. The inner NHR homotrimer is packed through the interactions of residues at the “*a*” and “*d*” positions (marked in yellow), whereas its residues at the “*e*” and “*g*” positions (in aquamarine) on the outside of the coiled-coil contact with the residues at the “*a*” and “*d*” positions (in red) of the CHR helices. Asn126 locates at the “*c*” position of CHR and is specifically marked. (**D**) Peptide sequences including various fusion inhibitors and the N36 and C34 derived from the Env of diverse HIV-1 isolates.

Human immunodeficiency virus type 1 (HIV-1) entry into target cells is mediated by its viral envelope (Env) glycoprotein, which is initially synthesized as a fusion-inactive precursor polypeptide (gp160) and then cleaved into the non-covalently surface subunit gp120 and transmembrane subunit gp41 [1,2]. As illustrated in Figure 1, the sequence structure of gp41 is defined with multiple functional domains, including the fusion peptide (FP), FP proximal polar region (FPPR), N-terminal heptad repeat (NHR), loop region, C-terminal heptad repeat (CHR), membrane proximal external region (MPER), transmembrane domain (TM), and cytoplasmic tail (CT). In a current fusion model, binding of gp120 to the cell receptor CD4 and a coreceptor (CCR5 or CXCR4) leads to a cascade of conformational changes in the Env complex that trigger the fusogenic activity of gp41. Firstly, the FP of gp41 is inserted into the cell membrane, leading an extended prehairpin state that transiently bridges the cell and viral membranes; then, three CHR helices fold in an antiparallel orientation into the trimeric NHR coiled coil grooves, resulting in a six-helix bundle (6-HB) structure that pulls the two membranes in apposition for fusion [3,4]. Two hydrophobic pockets in the C-terminal portion of the NHR helices play important roles for the stability of 6-HB, thus being considered ideal target sites for anti-HIV agents [2,5,6,7,8].

Peptides derived from both the NHR and CHR sequences of gp41 possess potent anti-HIV activity through binding to the gp41 prehairpin state, thus inhibiting 6-HB formation and membrane fusion in a dominant negative fashion [2,9]. In 2003, the CHR peptide T20 (enfuvirtide) was approved for combination therapy of the HIV-1 infection [10,11]; however, its clinical application has been significantly limited owing to a low genetic barrier to inducing drug resistance [12,13,14]. The responsible mutations for T20 resistance were consistently selected in vitro and in vivo, which mainly occurred in the inhibitor-binding sites on the NHR helices, with the amino acid Gly36-Leu45 stretch being a hotspot. Apart from the primary NHR mutations, some substitutions on the CHR helices also contributed to the T20 resistance as secondary or compensatory mutations, and in particular, a substitution of the polar residue asparagine to a positively charged residue lysine on position 126 (N126K) was frequently identified during the in vitro resistance selection and the treatment of HIV-1 infected individuals [10,13,14,15]. 

In succession to T20, a group of new HIV-1 fusion inhibitor peptides have been developed with improved antiviral activities and pharmaceutical properties [9,16]. In the studies, the CHR peptide C34 was widely used as a design template, because it has an N-terminal pocket-binding domain (PBD) and is considered a core CHR sequence. Indeed, the resulting C34 derivatives, such as SC34EK, sifuvirtide (SFT), and T2635, possessed significantly increased potencies in inhibiting both wild-type and T20-resistant HIV-1 isolates [17,18,19]. By adding the M-T hook structure to the PBD sequence, we previously generated several highly potent short-peptide fusion inhibitors, including MTSC22EK, HP23, and 2P23, which mainly target the deep pocket sites rather than the T20 resistance sites [20,21,22]. As noted, the resistance profiles of both the C34 derivatives and M-T hook-based short peptides were previously characterized by the in vitro selection of HIV-1 variants that were resistant to the inhibitors, and interestingly, the secondary N126K mutation was universally found to accompany with the diverse primary resistance mutations [23,24,25,26,27,28,29,30]. In the CHR sequence, Asn126 locates immediately at the downstream of the PBD sequence and it is a highly conserved residue among all the HIV-1, HIV-2, and even simian immunodeficiency virus (SIV) isolates (www.hiv.lanl.gov). Moreover, Asn126 also serves as a potential glycosylation site for gp41, but its functionality remains unclear. Therefore, here we focused to characterize the N126K mutation in the context of the HIV-1 resistance to various fusion inhibitors. 

## 2. Material and Methods

### 2.1. Cells and Plasmids

The following reagents were obtained through the AIDS Reagent Program, Division of AIDS, NIAID, NIH: TZM-bl cells stably expressing CD4 and CCR5 along with endogenously expressed CXCR4 from John C. Kappes and XiaoyunWu; the plasmid encoding the Env gp160 of HIV-1 92RW020 from the WHO Network for HIV isolation and characterization and Beatrice H. Hahn; the plasmid encoding the Env of HIV-1 REJO4541 from Beatrice H. Hahn and Jesus F. Salazar-Gonzalez; the plasmids encoding the Env of HIV-1 AC10.29 (SVPB13) and QH0692 (SVPB6) from David Montefiori and Feng Gao; the plasmids encoding the Env of HIV-1 ZM53M.PB12 (SVPC11) and ZM109F.PB4 (SVPC13) from Cynthia A. Derdeyn and Eric Hunter. The plasmid expressing DSP_1-7_ and 293FT cells stably expressing CXCR4/CCR5 and DSP_8-11_ were kindly provided by Zene Matsuda at the Institute of Medical Science of the University of Tokyo (Tokyo, Japan). HEK293T cells were purchased from the American type culture collection (ATCC) (Rockville, MD, USA). Cells were cultured in complete growth medium that consisted of Dulbecco’s minimal essential medium (DMEM) supplemented with 10% fetal bovine serum, 100 U/mL of Penicillin-Streptomycin, 2 mM L-Glutamine, and 1mM Sodium Pyruvate and were maintained at 37 °C in 5% CO_2_.

### 2.2. Peptide Synthesis

The fusion inhibitor peptides T20, sifuvirtide (SFT), SC22EK, MTSC22EK, and HP23, as well as the N36 and C34 peptides derived from the Env of divergent HIV-1 isolates were synthesized on rink amide 4-methylbenzhydrylamine (MBHA) resin by using a standard solid-phase 9-fluorenylmethoxy carbonyl (FMOC) method as described previously [31]. All the peptides were N-terminally acetylated and C-terminally amidated. They were purified by reverse-phase high-performance liquid chromatography (HPLC) to a purity of >95% and characterized for amino acid composition with mass spectrometry. Concentrations of the peptides were quantitated by UV absorbance and a theoretically calculated molar-extinction coefficient based on tryptophan and tyrosine residues.

### 2.3. Site-Directed Mutagenesis

N126K mutation was introduced into the Env glycoprotein gp160 of the HIV-1 isolates NL4-3, 92RW020, JRFL, REJO4541, AC10.29, QH0692, ZM53M.PB12, and ZM109F.PB4 according our protocol as described previously [31]. Briefly, two primers containing N126K occupied the same starting and ending positions on the opposite strands of a gp160-expressing plasmid. DNA synthesis was conducted by PCR in a 50-μL reaction volume using 100 ng of template plasmid, 10 pM upper and lower primers, and 5 U of the high-fidelity polymerase PrimeStar (TaKaRa, Dalian, China). PCR amplification was performed for one cycle of denaturation at 98 °C for 5 min, followed by 25 cycles of 98 °C for 15 s and 68 °C for 9 min, with a final extension at 72 °C for10 min. The amplicons were treated with restriction enzyme DpnI for 3 h at 37 °C, and DpnI-resistant molecules were recovered by transforming *Escherichia coli* strain DH 2Blue with antibiotic resistance. The required mutations were confirmed by DNA sequencing.

### 2.4. Single-Cycle Infection Assay

HIV-1 entry and its inhibition were determined by a single-cycle infection assay as described previously [31]. In brief, HIV-1 pseudovirus was generated by cotransfecting HEK293T cells with an Env-encoding plasmid and a viral backbone plasmid pSG3^△env^. Virus-containing supernatants were harvested 48 h after transfection, and 50% tissue culture infectious dose (TCID_50_) was measured in TZM-bl cells. The same amounts of pseudovirus particles were normalized by p24 antigen and their infectivity was determined in TZM-bl cells. To measure the inhibitory activity of various fusion inhibitors, peptides were prepared in 3-fold dilutions, mixed with 100 TCID_50_ of viruses. After incubation for 1 h at room temperature, the mixture was added to TZM-bl cells (10^4^ cells/well) and then incubated for 48 h at 37 °C. Luciferase activity was determined using luciferase assay reagents and a luminescence counter (Promega, Madison, WI, USA). Percent inhibition of the pseudovirus and 50% inhibitory concentration (IC_50_) of an inhibitor were calculated using GraphPad Prism software (GraphPad Software Inc., San Diego, CA, USA).

### 2.5. Cell-Cell Fusion Assay

A dual split-protein (DSP)-based fusion cell-cell assay was used to examine viral Env-mediated cell-cell fusion activity as described previously [31]. Briefly, a total of 1.5 × 10^4^ HEK293T cells (effector cells) were seeded on a 96-well plate and incubated overnight, and then they were transfected with a mixture of an Env-expressing plasmid and a DSP_1-7_ plasmid. After 24 h, 3 × 10^4^ 293FT cells expressing CXCR4/CCR5 and DSP_8-11_ (target cells) were resuspended in prewarmed culture medium that contains EnduRen live-cell substrate (Promega) at a final concentration of 17 ng/μL and then transferred to the effector cell wells at equal volumes. The mixed cells were spun down to maximize cell-cell contact, and the luciferase activity was measured as described above.

### 2.6. Capture ELISA

The expression and processing profile of HIV-1 gp160 were determined by a capture enzyme-linked immunosorbent assay (ELISA) as described [25]. Briefly, the wells of an ELISA plate were coated with a sheep anti-gp120 antibody (D7324) at 10 μg/mL and blocked by 3% bovine serum albumin (BSA). Cell lysates or culture supernatants of Env-transfected cells were added to the wells and incubated at 37 °C for 1 h. After five washes with PBS-Tween, 50 μL of the human anti-gp120 monoclonal antibody VRC01 or anti-gp41 monoclonal antibody 10E8 (5 μg/mL) was added and incubated at 37 °C for 1 h. The bound antibodies were then detected by horse-radish peroxidase (HRP)-conjugated goat anti-human IgG and 3,3,5,5-tetramethylbenzidine at a absorbance of *A*450.

### 2.7. Western Blotting Assay

The expression and processing profile of HIV-1 gp160 were also examined by Western blotting assay as described previously [31]. In brief, HEK293T cells were transfected with an Env-expressing plasmid and cultured for 48 h. Then, the lysates of transfected cells were centrifuged at 20,000× *g* at 4 °C for 15 min to remove insoluble materials. Equal amounts of total proteins were separated by SDS-PAGE and then transferred to a nitrocellulose membrane, followed by blocking with 5% nonfat dry milk solution in Tris-buffered saline (TBS, pH 7.4) at room temperature for 1 h. The membrane was incubated with a rabbit anti-gp120 polyclonal antibody (SinoBiological, Beijing, China) or the human anti-gp41 monoclonal antibody 10E8 overnight at 4°C. After washing three times with TBS-Tween 20, the membrane was incubated with IRDye 680LT goat-anti-rabbit IgG or IRDye 800CW goat-anti-human IgG for 2 h at room temperature. As an internal control, *β*-actin was detected with a mouse anti-β-actin monoclonal antibody (Sigma, St. Louis, MO, USA) and IRDye 680LT donkey-anti-mouse IgG. The membrane was then scanned using the Odyssey infrared imaging system (LI-COR Biosciences, Lincoln, NE, USA). 

To analyze HIV-1 Env incorporation and cleavage in pseudotype particles, cell culture supernatants containing virions were precipitated with 3% PEG-6000 at 4 °C for 2 h, and then concentrated by centrifugation at 14,000× *g* at 4 °C for 60 min. The purified viral particles were re-suspended in RIPA lysis buffer supplemented with protease inhibitor cocktail (Sigma) and subjected to SDS-PAGE and immunoblotting as described above.

### 2.8. Flow Cytometry Assay

Cell surface expression of wild-type or mutant Envs was detected by flow cytometry. Briefly, HEK293T cells (2 × 10^5^) were seeded in 24-well plates and incubated for 12 h, followed by transfection of plasmids encoding viral Env glycoproteins. The transfected cells were harvested at 36 h after transfection, washed two times with PBS, and then incubated with VRC01 antibody at 4 °C for 1 h. After two washes with PBS, cells were incubated with DyLight^®^488 labeled-rabbit anti-human antibody (Abcam, Cambridge, MA, USA) at 4 °C for 1 h. After three washes, cells were re-suspended in PBS and analyzed by FACSCantoII instrument (Becton Dickinson, Mountain View, CA, USA).

### 2.9. Circular Dichroism (CD) Spectroscopy

CD spectroscopy was performed to determine the α-helicity and thermostability of the peptide complexes as described previously [25]. Briefly, the NHR peptide N36 was incubated with an equal molar concentration of the CHR peptide C34 or its N126K mutant at 37 °C for 30 min in phosphate-buffered saline (PBS, pH 7.2). CD spectra were acquired on a Jasco spectropolarimeter (model J-815) using a 1-nm bandwidth with a 1-nm step resolution from 195 to 260 nm at 20 °C. Spectra were corrected by subtraction of a solvent blank. The α-helical content was calculated from the CD signal by dividing the mean residue ellipticity ([θ]) at 222 nm by the value expected for 100% helix formation (−33,000 degrees cm^2^ dmol^−1^). Thermal denaturation was conducted by monitoring the ellipticity change at 222 nm from 20 to 98 °C at a rate of 2 °C/min using a temperature controller, and melting temperature (*T*_m_) was defined as the midpoint of the thermal unfolding transition.

### 2.10. Isothermal Titration Calorimetry (ITC)

The interaction affinity between N36 and C34 or its N126K mutant peptide was determined by ITC experiment as described previously [32]. Thermodynamic parameters were acquired on an ITC-200 Microcalorimeter instrument (MicroCal, Northampton, Massachusetts, USA). Briefly, 1 mM of N36 was dissolved in double distilled H_2_O and injected into a chamber containing 100 μM of C34 or its N126K mutant peptide. The time between injections was 240 s and the stirring speed was 400 rpm. The experiments were carried out at 25 °C. Data acquisition and analysis were done using MicroCal Origin software (version 7.0).

### 2.11. Crystallization and Structure Determination

Equal amounts of N36 and C34_N126K_ peptides (1:1 molar ratio) were mixed in a denaturing buffer (100 mM NaH_2_PO_4_, 10 mM Tris-HCl, pH 8.0, 8 M urea). The mixture was dialyzed against the buffer containing 50 mM Tris-HCl, pH 8.0, 100 mM NaCl at 4°C overnight to allow refolding. The resulting sample was then subjected to size-exclusion chromatography with Superdex-75 10/300 GL (GE Healthcare, Piscataway, NJ, USA). The predominant peak corresponding to the 6-HB size was collected. The 6-HB containing N36 and C34_N126K_ was crystallized by mixing equal volumes of the purified sample (10 mg/mL) and reservoir solution containing 0.1 M ammonium acetate, 0.1 M Bis-Tris (pH 5.8), and 21% (*w*/*v*) polyethylene glycol (PEG)-10000. The cryocooling was achieved by soaking the crystals 30–60 s in a reservoir solution containing 15% glycerol followed by flash freezing in liquid nitrogen. Complete datasets were collected on beamline BL19U at the Shanghai Synchrotron Research Facility (SSRF) with x-ray wavelength of 0.98 Å. The crystal belonged to the space group of C121, contained a complete 6-HB per asymmetry unit, and diffracted the x-ray to the resolution limit of 1.65 Å. The structure of 6-HB was solved by molecular replacement (Phaser for MR, CCP4 package) using the crystal structure of N36/C34 (Protein Data Bank [PDB] code 1AIK) as a searching model. The coordinates were deposited in the PDB under accession number 6KTS. The structure was refined using PHENIX resulting in a final atomic model with excellent refinement statistics and stereochemistry qualities (Table 1). The structure was validated by MolProbity analysis. The MolProbity score for the crystal structure of N36/C34_N126K_ is 1.49, rating 91st percentile among structures of comparable resolution. The Ramachandran plot finds all residues in the favored area.

## 3. Results

### 3.1. Single N126K Mutation-Mediated Resistance Profiles in Divergent HIV-1 Isolates

In the previous studies [23,24,25,26,27,28,29,30], laboratory-adapted HIV-1 strains were exclusively used to characterize the resistance profiles for various fusion inhibitors, and in which the secondary N126K mutation was universally identified. In order to verify its biological relevance, here we generated a single N126K mutation on a large panel of HIV-1 Envs with divergent subtypes and phenotypes, including NL4-3 (subtype B, CXCR4-tropic), 92RW020 (subtype A, CCR5-tropic), JRFL (subtype B, CCR5-tropic), REJO4541 (subtype B, CCR5-tropic), AC10.29 (subtype B, CCR5-tropic), QH0692 (subtype B, CCR5-tropic), ZM53M.PB12 (subtype C, CCR5-tropic), and ZM109F.PB4 (subtype C, CCR5-tropic). Then, the corresponding Env pseudotyped viruses were generated to determine the effect of the introduced N126K substitution on the sensitivity of diverse HIV-1 isolates to several fusion inhibitors, including T20, C34, SFT, SC22EK, MTSC22EK, and HP23. As shown in Table 2, the fold changes (FC) of the IC_50_ values reflect the resistance properties of the N126K mutant viruses relative to the wild-type (WT) viruses. Consistent to the previous results, the laboratory-adapted CXCR4-tropic virus NL4-3 with N126K (NL4-3_N126K_) mediated mild cross-resistance to various fusion inhibitors; however, seven primary CCR5-tropic viruses displayed different resistance profiles. It is worth to note that two mutants, 92RW020_N126K_ and JRFL_N126K_, could confer relatively higher resistance, with the fold changes of 7.8 and 9.9, respectively.

### 3.2. Effect of N126 Mutation on the Functionality of Divergent HIV-1 Envs

**Figure 2:**
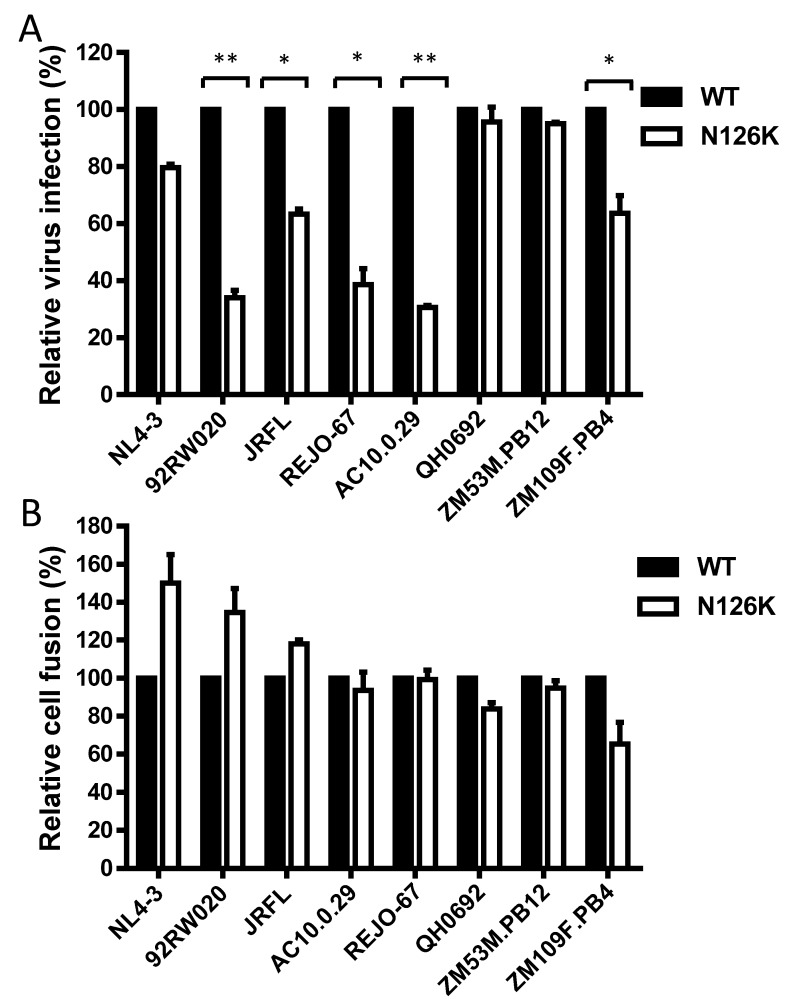
Effects of the N126K mutation on the functionality of HIV-1 Env. (**A**) Relative infectivity of wild-type (WT) and N126K mutant pseudoviruses in TZM-bl cells was determined by a single-cycle infection assay. The WT and mutant pseudoviruses were normalized to a fixed amount by p24 antigen. The luciferase activity of WT virus was treated as 100%, and the relative infectivity of mutant virus was calculated accordingly. (**B**) Relative fusion activity of WT and mutant HIV-1 Envs was determined by a dual split-protein (DSP)-based assay. HEK293T cells cotransfected with viral Env and DSP_1-7_ were used as effector cells, and 293FT cells stably expressing CXCR4/CCR5 and DSP_8-11_ were used as target cells. Similarly, the luciferase activity of WT Env was treated as 100%, and the relative activity of mutant Env was calculated accordingly. For both viral entry and fusion, data were derived from the results of three independent experiments and are expressed as mean and standard deviation. A *t* test was performed to compare the WT and mutants; * and ** express *p* < 0.05 and *p* < 0.01, respectively.

**Figure 3:**
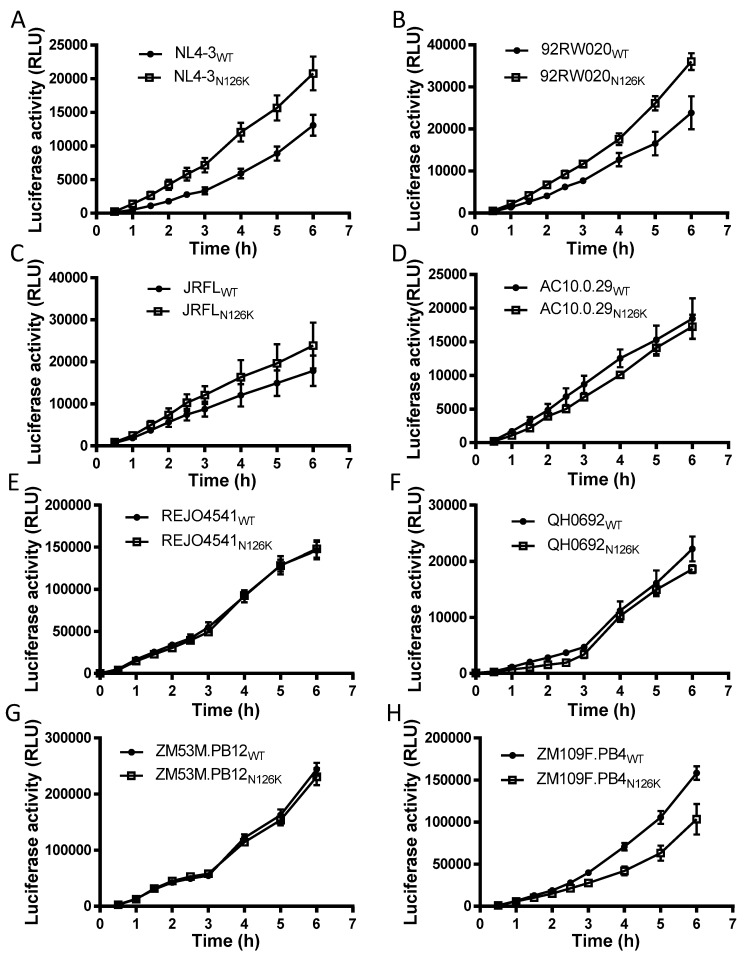
Kinetics of diverse HIV-1 Env-mediated cell-cell fusion determined by DSP-based assay. The fusion activities of viral Envs derived from HIV-1 NL4-3 (**A**), 92RW020 (**B**), JRFL (**C**), REJO4541 (**D**), AC10.29 (**E**), QH0692 (**F**), ZM53M.PB12 (**G**), and ZM109F.PB4 (**H**) were respectively measured at different time points, and the fusion kinetics of wild-type (WT) and its N126K mutant Envs was compared directly. Data were derived from the results of three independent experiments and are expressed as means ± standard deviations (SD).

We sought to determine the effect of N126K mutation on the functionality of viral Env. Firstly, the infectivity of HIV-1 pseudoviruses carrying wild-type (WT) and N126K Envs was measured by a single-cycle infection assay, in which the entry efficiency in TZM-bl cells of a WT virus was normalized to 100% and the relative infectivity of its N126K mutant virus was calculated accordingly. As shown in Figure 2A, the N126K mutation exerted different actions in divergent pseudoviruses, with a significantly reduced infectivity in 92RW020, JRFL, REJO4541, AC10.29, and ZM109F.PB4, but not in NL4-3, QH0692, and ZM53M.PB12. Next, we compared the activities of diverse WT and mutant Envs to mediate cell-cell fusion by a dual split-protein (DSP)-based fusion assay. It was found that none of the N126K mutants exhibited a significant difference compared to the fusion activity of its WT Env (Figure 2B). When the fusion activities of the WT and N126K Envs were measured at different time points, the results supported the observation above (Figure 3).

### 3.3. N126K Mutation Does Not Affect the Expression of Viral Env Glycoprotein but Causes Deglycosylation in Gp41

**Figure 4:**
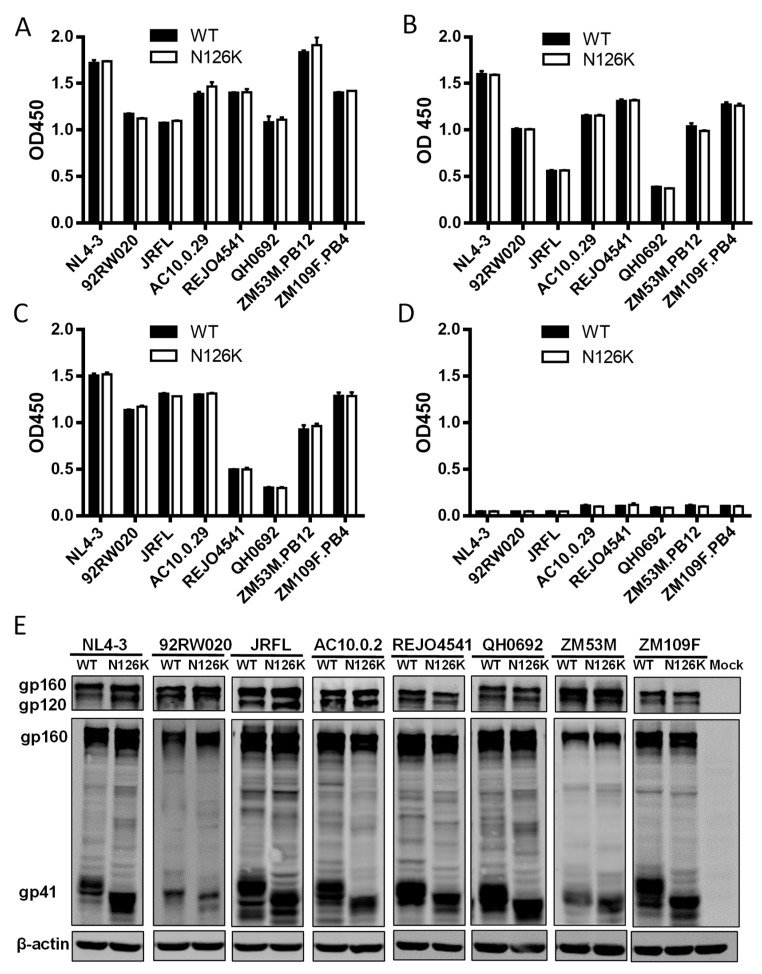
Effects of the N126K mutation on the expression and processing of HIV-1 Env glycoprotein. HEK293T cells were transfected by plasmids encoding wild-type (WT) or N126K mutant Envs. The viral Env glycoproteins (gp160/gp120/gp41) in the lysates of transfected cells (**A**,**B**) or culture supernatant (**C**,**D**) were detected by capture ELISA, in which the sheep anti-gp120 antibody D7324 was used as a capture antibody and the human anti-gp120 monoclonal antibody VRC01 (**A**,**C**) and anti-gp41 monoclonal antibody10E8 (**B**,**D**) were used as probes. (**E**) The viral Env glycoproteins in the lysates of transfected cells were detected by Western blot with a rabbit anti-gp120 polyclonal antibody (upper panel) and 10E8 (middle panel). The *β*-actin protein was detected as an internal control (lower panel). The reaction bands corresponding to gp160, gp120, and gp41 are respectively marked. The experiments were repeated three times, and representative data are shown.

We next focused on characterizing the effect of N126K mutation on the expression and processing of viral Env. Firstly, a capture ELISA-based method with the human anti-gp120 monoclonal antibody VRC01 and anti-gp41 monoclonal antibody 10E8 was applied as described previously [23,25,26]. It is known that VRC01 can react with both the uncleaved gp160 and cleaved gp120 proteins in the lysates of transfected cells and with the secreted gp120 protein in the cell culture supernatants, whereas 10E8 reacts only with the gp160 and cleaved gp41 in the cell lysates but not with gp120 in the culture supernatants, thus their reactivity can theoretically reflect the expression profiles of gp160/gp120/gp41. As shown in Figure 4A,B, both VRC01 and 10E8 detected similar levels of the proteins in the lysates of HEK293T cells transfected with the WT and mutant Env-expressing plasmids. Simultaneously, VRC01 reacted equivalently with the cell culture supernatants, while 10E8 had no reaction with the supernatants as expected (Figure 4C,D). 

**Figure 5:**
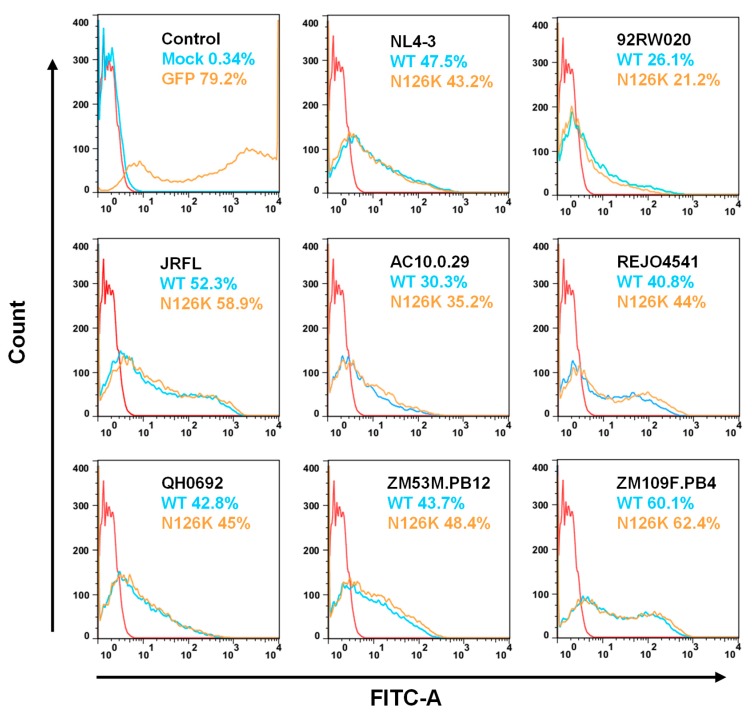
Expression of WT and mutant Env glycoproteins on the surface of transfected cells. The viral Env glycoproteins expressed on the surface of transfected HEK293T cells were detected with the human anti-gp120 antibody VRC01 by flow cytometry. The experiments were performed three times, and representative data are shown. The *red control curve* was reused in the *panels*.

**Figure 6:**
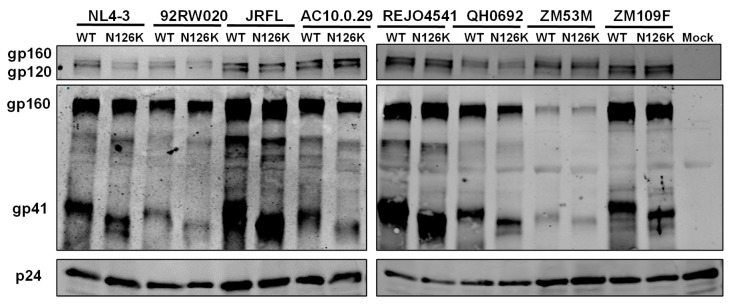
HIV-1 Env incorporation in pseudotype particles determined by immunoblotting. Concentrated virions were re-suspended in RIPA lysis buffer and subjected to SDS-PAGE and Western blot, in which a rabbit anti-gp120 polyclonal antibody (upper panel), human anti-gp41 antibody 10E8 (middle panel), and a rabbit anti-HIV P24 polyclonal antibody (lower panel) were respectively used.

The expression and processing of the Env glycoproteins were further characterized by Western blotting assay, in which a rabbit anti-gp120 polyclonal antibody and 10E8 were respectively used to probe the cleaved and uncleaved Env glycoproteins. The results verified that the WT and N126K mutant showed similar expression and processing pattern of gp160 in all of eight viral Envs (Figure 4E). In a line with our previous results in NL4-3 Env [25], N126K could abolish the glycosylation site of viral Envs thus resulting in a reduced size of gp41 protein. The cell surface expression of viral Envs was also analyzed by flow cytometry, demonstrating that each N126K mutants had a comparable expression level with its WT Env (Figure 5). Moreover, we determined whether the N126K mutation affected HIV-1 Env incorporation in the pseudotype particles. As shown by Western blot (Figure 6), the WT and N126K mutant Envs displayed similar expression levels and cleavage patterns either. Taken these results together, we conclude that the N126K mutation does not interfere with the expression and processing of viral Env glycoprotein, but it can result in gp41 with deglycosylation.

### 3.4. N126K Mutation Can Significantly Increases the Thermostability of 6-HB Structure

**Figure 7:**
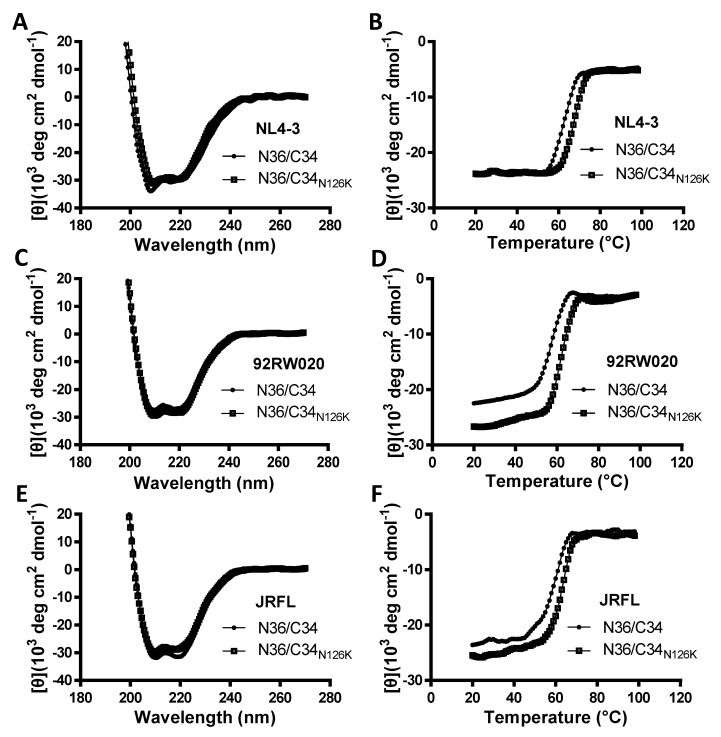
Effects of the N126K mutation on the secondary structure and thermostability of 6-HB conformation. The α-helicity and thermostability of the complexes formed by the NHR peptide N36 and CHR peptide C34 or its N126K mutant (C34_N126K_) derived from HIV-1 NL4-3 (**A**,**B**), 92RW020 (**C**,**D**), and JRFL (**E**,**F**) were determined by CD spectroscopy. The final concentration of each peptide was 10 μM in PBS. The experiments were repeated three times, and representative data are shown.

**Figure 8:**
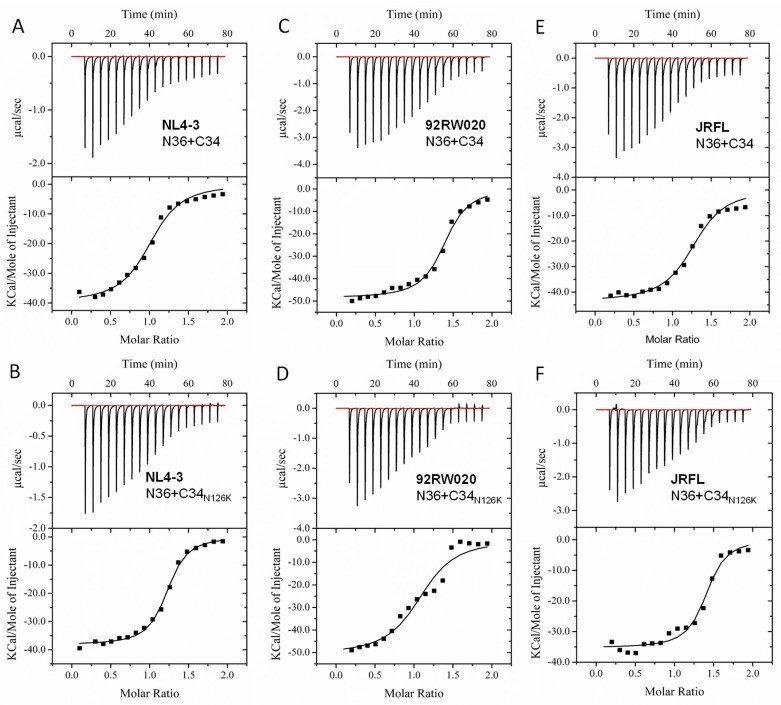
Effects of the N126K mutation on the interaction of the NHR and CHR peptides. Thermodynamic profiles of the molecular interactions between the N36 and C34 or C34_N126K_ peptides of HIV-1 NL4-3 (**A**,**B**), 92RW020 (**C**,**D**), and JRFL (**E**,**F**) were measured by ITC. The titration traces are shown at the top, and the binding affinities are shown at the bottom. The experiments were repeated three times, and representative data are shown.

The NHR-derived peptide N36 and CHR-derived peptide C34 were initially used to determine the crystal structure of 6-HB thus being considered a core helical sequence of gp41 [4]. We previously found that N126K mutation could significantly increase the thermostability of 6-HB structure modeled by the NL4-3 Env-derived N36 and C34 peptides [25]. In this study, we synthesized a panel of N36 and C34 peptides corresponding to the Env sequences of NL4-3, 92RW020, and JRFL, as illustrated in Figure 1D. The effect of N126K mutation on the α-helicity and thermostability of 6-HB was further analyzed by circular dichroism (CD) spectroscopy. As shown in Figure 7 and Table 3, the N126K mutation did not affect the α-helicity of each 6-HBs; however, it consistently enhanced their thermal stabilities. Specifically, the N36/C34_N126K_-based 6-HBs of NL4-3, 92RW020, and JRFL exhibited increased *T*_m_ values of 4.7, 5.3, and 4.8 °C as compared to the wild-type N36/C34-based ones, respectively. We also analyzed the interaction between the wild-type and mutant peptides by isothermal titration calorimetry (ITC), which measures the thermodynamic parameters of the peptide pairs that reflect an instant molecular interaction, whereas the CD spectroscopy measured the α-helicity and thermostability of a preformed peptide complex. Figure 8 and Table 3 show the results, including the reaction stoichiometric (*N*), binding constant (*K*), enthalpy (Δ*H*), and entropy (Δ*S*). Obviously, the interaction of N36 and C34 peptides in the presence and absence of N126K was a typical enthalpy-driven reaction, in which plenty of heat was released, and the N126K mutation could slightly increase the binding constant of C34 peptides.

### 3.5. Structural Basis of N126K Mutation in 6-HB

**Figure 9:**
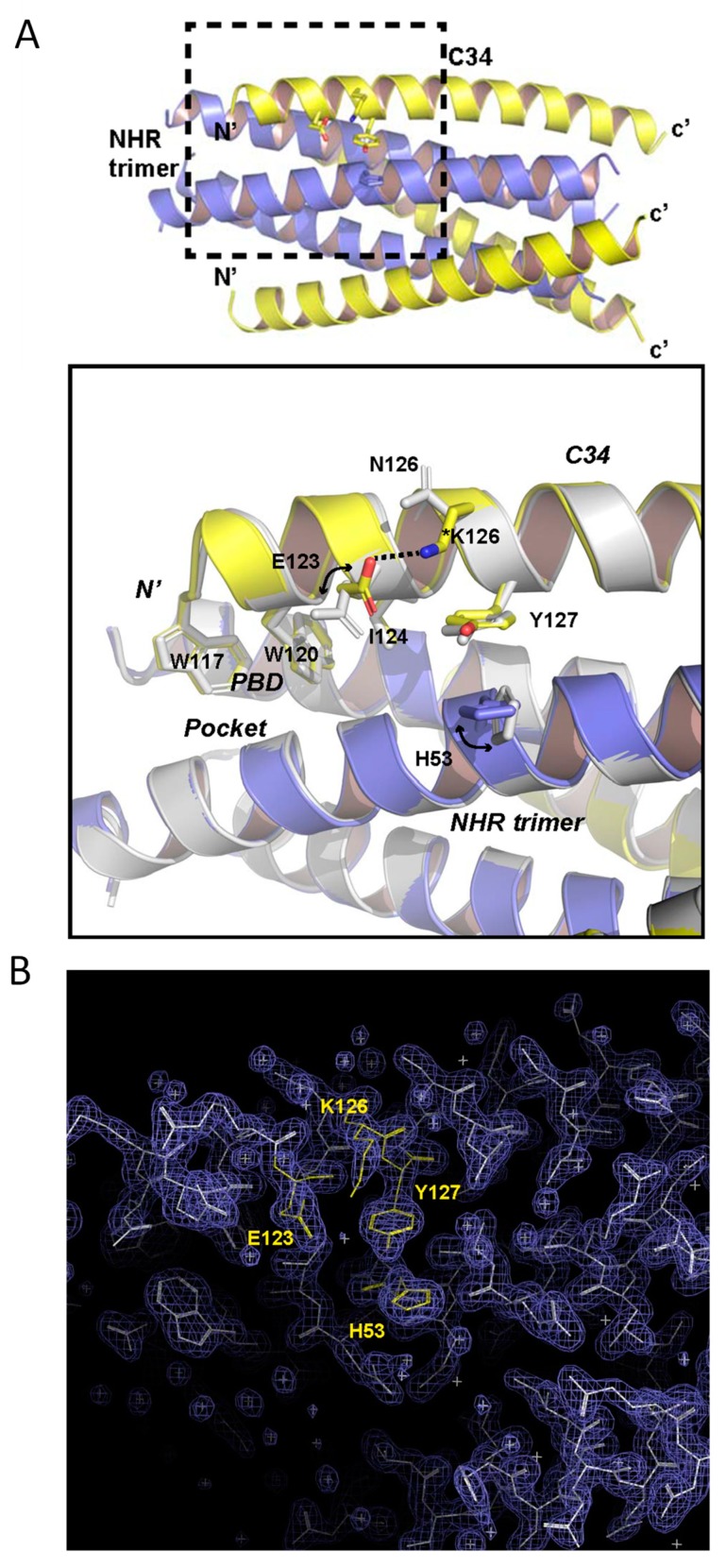
Structural basis for the N126K mutation-mediated stabilization 6-HB conformation. (**A**) Upper part, an overview 6-HB structure comprising of N36/C34_N126K_ peptides derived from HIV-1 strain NL4-3. N36 is colored in blue, C34_N126K_ is colored in yellow. The dashed line box area highlights the site of conformational changes associated with N126K mutation, which is magnified below. Lower part, magnified view of the structural superimposition of the N36/C34_N126K_ structure with the N36/C34 structure colored in gray (PDB: 1AIK). Residues involved in conformational changes and residues forming the PBD domain are shown with stick models. (**B**) Final 2Fo-Fc map (contour 1.5s) of N36/C34_N126K_ crystal structure with superimposed model. Residues K126, E123, Y127 and H53 involved in side chain conformation changes between C34 and C34_N126K_ are colored in yellow and labelled.

In order to reveal the structural basis underlying the N126K phenotype, we determined the crystal structure of the C34_N126K_ and N36 peptide complex. As shown in Figure 9A, two peptides form a canonical 6-HB structure, in which three C34_N126K_ peptides (CHR) bind to the central trimeric N36 coiled coil (NHR) in an antiparallel orientation. All residues of C34_N126K_ stands out clearly in a final electron density map, indicating 6-HB containing C34_N126K_ is highly stable. To reveal the impact of N126K mutation, we superimposed 6-HB structure containing the wild-type C34 (PDB ID: 1AIK) with our structure. Two structures are well aligned, which is indicated by nearly identical conformations adopted by PBD residues W117, W120 and I124 from C34 and C34_N126K_. The conformation of residue E123 of C34 in 1AIK is obviously detrimental to C-peptide binding. Because (a), the hydrophilic and highly charged side chain of E123 is positioned too closed to the hydrophobic pocket below, where it may destabilize the hydrophobic interaction between PBD and the pocket. (b), the side chains of H53 does not orient parallelly with the aromatic side chain of Y127 in 1AIK, suggesting they do not form an optimal π-stacking interaction. It is possible that E123 side chain is positioned in the proximity of H53 side chain, hence the electrostatic attraction between E123 and H53 interferes with π-stacking between H53 and Y127. In sharp contrast, our structure demonstrates that when N126 was replaced with a positively charged lysine, residue K126 attracted the negatively charged side chain of E123 and tilted its side chain away from the hydrophobic pocket, hence the interaction between PBD and the hydrophobic pocket could be further stabilized. Subsequently, we found that H53 side chain shifted to a conformation parallelly to the side chain of Y127, indicating the π-stacking between H53 and Y127 restored. In addition, the distance between carboxylate side chain of E123 and Nζ atom of K126 is 2.9Å, indicating a tight hydrogen bond interaction. This hydrogen bond plays a role of stabilizing the helical conformation of N-terminal portion of C34_N126K_, thus favors the binding of the C-peptide to NHR trimer. All residues (K126, E123, Y127, and H53) involved in side chain conformation changes described above are clearly visible in the final electron density map (Figure 9B).

## 4. Discussion

In the context of the secondary mutation N126K in the CHR of HIV-1 gp41 was commonly observed in the selection of mutant viruses resistant to various fusion inhibitors, here we specifically characterized its biological relevance and structural properties by multiple approaches. A panel of mutant HIV-1 psedoviruses was generated, which verified that a single N126K mutation could confer certain degrees of resistance and cross-resistance associated with the specificities of viral strains and fusion inhibitor peptides. We found that most of the HIV-1 psedoviruses bearing the N126K mutation had significantly decreased infectivity in terms of the single-cycle cell entry efficiency, but their Envs maintained a similar activity to medicate cell-cell fusion; the N126K mutation did not affect the expression and processing of viral Env glycoprotein, but it disrupted the Asn126-mediated glycosylation site in gp41. The biophysical data verified that the N126K mutation could enhance the thermal stability of 6-HB structure modeled by the NHR and CHR peptides. Finally, we determined the crystal structure of 6-HB with C34 bearing the N126K mutation, which revealed the interhelical and intrahelical interactions underlying the increased thermostability. Taken together, the present studies inform the functional and structural changes caused by the N126K mutation and would help our understanding on the mechanism of HIV-1 Env-mediated membrane fusion and its resistance mode to the fusion inhibitors.

Refolding of the gp41 NHR and CHR into a 6-HB conformation drives the viral and cellular membranes in close apposition and releases energy to overcome the kinetic barrier required for virus-cell membrane fusion. A body of evidence demonstrates that HIV-1 resistance to various peptide-based fusion inhibitors is mediated by genetic mutations in the NHR sequence, especially within the inhibitor-binding sites [13,15,23,24,25,26,27,28,29,33,34]. Notably, some CHR mutations were frequently observed during escape selection and in T20-containing clinical therapy, such as N126K, E136G, E137K, S138A, and E154K [27,28,29,30,33,35,36,37,38,39,40,41,42]. While other CHR mutations were often induced by a specific peptide, the N126K mutation was routinely selected by various fusion inhibitors. In this regard, we previously characterized the resistance profiles of the C34 derivatives SFT and SC29EK as well as the small inhibitors SC22EK and MTSC22EK, and all the resistant HIV-1 mutant viruses developed the N126K mutation [23,24,25,26]. For the primary NHR mutations, Eggink and coworkers described several resistance pathways, including large amino acid-mediated steric obstruction, small amino acid-mediated reduced contact, basic amino acid-mediated electrostatic attraction, and acidic amino acid-mediated electrostatic repulsion [43]; we also proposed the other two NHR-mediated resistance pathways: the disruption of hydrogen bonds and hydrophobic contacts would severely impair the binding of inhibitors thus determining the resistance [44]. For the CHR mutations, it has been considered that a secondary mutation can compensate the delayed membrane fusion kinetics caused by primary NHR mutations [40,45]. Several studies also described that the secondary CHR mutations can markedly improve the thermal stability and/or free energy of viral 6-HB structure [15,46,47,48]. With three pairs of N36/C34 peptides derived from divergent HIV-1 isolates, here we verified again that a single N126K mutation did significantly stabilize the 6-HB conformation. As illustrated in the helical wheel model (Figure 1C), Asn126 is located outside the binding surface of a CHR peptide, we wondered how its substitution with a positively charged residue affected the interactions between the NHR and CHR helices. To this, the presented crystal structure of 6-HB emphasizes the critical roles of the N126K mutation in the interactions. Herein, we summarize that as follows: (1) N126K changes the conformation of surrounding amino acid residues and introduces an intrahelical salt bridge between Glu123 and Lys126, thus improving the helical stability of CHR itself; (2) a stabilized Glu123 further stabilizes the hydrophobic pocket site of 6-HB; (3) π-stacking between Tyr127 and His53 associates the NHR and CHR helices. 

A number of studies demonstrate that the secondary mutations in the CHR of gp41can render the virus with mild resistance to the fusion inhibitors and boost the resistance level when combined with the primary NHR mutations [13,15,23,24,25,26,27,28,29,30,33,34,35,36,37,38,39,40,41,42]. However, all the previous works were focused on a single HIV-1 strain or peptide. In this study, we generated a large panel of HIV-1 mutants with diverse subtypes and phenotypes and evaluated a group of representative fusion inhibitors. Apparently, the N126K mutation-mediated resistance profiles differed between the viruses and/or inhibitors (Table 2). For instance, the 92RW020_N126K_ and JRFL_N126K_ mutants were able to confer moderate resistance to T20, but only with mild or no resistance to C34 and the deep pocket-targeting short-peptide inhibitors. In contrast, the N126K mutant viruses generally had minor resistance to the M-T hook structure modified fusion inhibitors (MTSC22EK and HP23), which confirmed our previous observations [20,21,22,49,50]. We also found that the N126K mutation affected the functionalities of viral Env to mediate cell entry and cell-cell fusion differently. The mechanisms underlying such disparities in the resistance profile and infectivity need to be further characterized in details. As mentioned, Asn126 is a highly conserved residue among all the HIV-1 isolates. And similarly, the Asn126-based glycosylation site is also evolutionarily conservative; however, its exact functions in the gp41-dependent membrane fusion and viral escape to the fusion inhibitors are largely unknown. A previous study showed that the removal of the Asn126-linked glycan enhanced the thermal stability of the NHR-CHR complex, which might be deduced in viral 6-HB structure [51]. The present studies verified that the N126K mutation could result in the loss of the glycosylation site in the gp41 proteins from all of eight HIV-1 isolates. Definitely, we are interested to characterize such phenotypes in future studies. 

## Figures and Tables

**Table 1:** Crystallographic data collection and refinement statistics.

Parameter	N36/C34_N126K_
**Data collection**	
Space group	C121
Cell dimensions	
*a,b,c*	88.86 Å 50.81 Å 56.11 Å
*α,β,γ*	90.00° 90.40° 90.00°
X-ray source	SSRF BEAMLINE BL19U1
Wavelength	0.98 Å
Data range	50–1.65 (1.75–1.65) Å
Reflections unique	29,914
*R_merge_* (%)	5.3 (83.3)
*I/σI*	17.79 (1.94)
Completeness (last shell)	98.9% (97.9%)
Redundancy (last shell)	6.54 (6.23)
**Refinement**	
Resolution range	44.11–1.65 (1.70–1.65) Å
No.Reflections	29,894
*R _work_^a^/R _free_^b^*	0.2062/0.2258
Nonhydrogen atoms	1987
Protein	1785
Water	202
*N*-Acetyl group	6
B-Factor averages	34.0 Å^2^
Root mean square deviation	
Bond length	0.025 Å
Bond angles	1.35°
**Validation**	
MolProbity score	1.49, rating 91st percentile among structures of comparable resolution
% Favored regions and Outliers in Ramachandran plot	100.0, 0.0, 0.0

^a^*R*_work_ indicates ∑_hkl_‖*F*_obs_ *(hkl)*∣-∣*F*_calc_(hkl)‖/∑*_hkl_*∣*F*_obs_(hkl)∣. ^b^ *R*_free_ indicates the cross-validation *R* factor for 5% of reflections against which the model was not refined.

**Table 2:** Effect of the N126K mutation on the susceptibility of divergent HIV-1 subtypes to peptide fusion inhibitors ^a^.

HIV-1	Subtype	T20		C34		SFT		SC22EK		MTSC22EK		HP23	
		IC_50_ (nM)	FC	IC_50_ (nM)	FC	IC_50_ (nM)	FC	IC_50_ (nM)	FC	IC_50_ (nM)	FC	IC_50_ (nM)	FC
NL4-3_WT_	B	71± 13.8		1.5 ± 0.4		1.9 ± 0.8		47.9 ± 12.9		2 ± 0.8		0.6 ± 0.2	
NL4-3_N126K_	B	153.4 ± 2.2	2.2	5.7 ± 2	3.8	5.1 ± 1	2.7	86.5 ± 1.8	1.8	4.9 ± 1.6	2.5	1.5 ± 0.2	2.6
92RW020_WT_	A	16.3 ± 6.2		4.8 ± 0.5		7.5 ± 3.1		427.7 ± 56.9		6.7 ± 2.8		2.1 ± 1	
92RW020_N126K_	A	126.8 ± 124.1	7.8	6.2 ± 0.9	1.3	10.9 ± 1.3	1.5	1333.5 ± 766.2	3.1	14.3 ± 6.8	2.1	3.3 ± 1	1.5
JRFL_WT_	B	13.9 ± 2.1		5.8 ± 2.4		9 ± 2.1		467.2 ± 104.6		19.5 ± 6.6		5.9 ± 2	
JRFL_N126K_	B	137.4 ± 52.6	9.9	27.9 ± 16.5	4.8	31.3 ± 12.9	3.5	600.4 ± 93.3	1.3	57.4 ± 18.4	3	15.3 ± 0.2	2.6
REJO4541_WT_	B	19 ± 1.7		9.9 ± 4.4		6.1 ± 0.2		381.7 ± 95.5		9.6 ± 2.5		3.1 ± 0.5	
REJO4541_N126K_	B	66.9 ± 14	3.5	15 ± 13.8	1.5	10.5 ± 1.9	1.7	1284 ± 78.2	3.4	13.2 ± 3.8	1.4	5.7 ± 2	1.8
AC10.29_WT_	B	13.2 ± 7.1		1.4 ± 0.2		2.1 ± 1		110.9 ± 37.1		3.1 ± 0.7		0.7 ± 0.3	
AC10.29_N126K_	B	36.4 ± 1.4	2.8	1.8 ± 0.3	1.3	2.5 ± 0.9	1.2	247.7 ± 38.7	2.2	4.1 ± 1.6	1.3	1.2 ± 0	1.6
QH0692_WT_	B	92.1 ± 19.9		59.1 ± 8.4		28.6 ± 1.5		2055 ± 291.9		68.9 ± 7.5		34.6 ± 4.9	
QH0692_N126K_	B	184.9 ± 44.5	2	32.3 ± 4.9	0.6	62.5 ± 7.7	2.2	1163.3 ± 202.7	0.6	92.2 ± 22.4	1.3	41.9 ± 3	1.2
ZM53M.PB12_WT_	C	21.4 ± 10.8		10.7 ± 6.2		2.1 ± 0.8		47.3 ± 3.4		2.7 ± 0.2		1.2 ± 0.1	
ZM53M.PB12_N126K_	C	49.8 ± 7.8	2.3	15.7 ± 3.3	1.5	2.8 ± 0.9	1.3	164.5 ± 18.7	3.5	3.2 ± 0.7	1.2	1.6 ± 0.3	1.3
ZM109F.PB4_WT_	C	12.9 ± 3.5		1.5 ± 0.3		2.6 ± 1.1		43.6 ± 8.1		2 ± 0.4		0.8 ± 0.1	
ZM109F.PB4_N126K_	C	23.8 ± 7.5	1.9	2.8 ± 0.5	1.8	4.5 ± 0.4	1.7	79.4 ± 6.9	1.8	3.3 ± 0.4	1.6	1.4 ± 0.3	1.8

^a^ Data were derived from three independent experiments and express as means ± standard deviations (S.D.). Fold change (FC) in the IC_50_ was determined relative to the WT level.

**Table 3:** Biophysical characterization of the N126K mutation on the interaction of the NHR and CHR helices ^a^.

Peptide Pair	CD Data		ITC Data			
	Helix (%)	*T*_m_ (°C)	*N* (Site)	*K* (M^-1^)	Δ*H* (cal/mol)	ΔS (cal/mol/deg)
NL4-3 N36/C34	94.7 ± 3.7	63.8 ± 0.7	1	6.4 × 10^5^ ± 1.2 × 10^5^	−4 × 10^4^ ± 1059	−112.5 ± 9.2
NL4-3 N36/C34_N126K_	91.2 ± 3.8	68.5 ± 0.8	1.2	1.9 × 10^6^ ± 2.4 × 10^5^	−3.8 × 10^4^ ± 427.5	−94.5 ± 6.9
92RW020 N36/C34	79.1 ± 4.1	57.4 ± 0.8	1.4	9.4 × 10^5^ ± 1.8 × 10^5^	−4.9 × 10^4^ ± 787.7	−121.6 ± 14.2
92RW020 N36/C34_N126K_	78.8 ± 3.7	62.7 ± 0.6	1	1.3 × 10^6^ ± 5 × 10^5^	−5.5 × 10^4^ ± 1862	−151.5 ± 20.4
JRFL N36/C34	82.3 ± 6.1	57.9 ± 0.8	1.3	4.3 × 10^5^ ± 8.7 × 10^4^	−4.4 × 10^4^ ± 1005	−123.5 ± 10
JRFL N36/C34_N126K_	82.8 ± 4.9	62.7 ± 0.7	1.4	1.2 × 10^6^ ± 3.4 × 10^5^	−3.5 × 10^4^ ± 748.6	−92.9 ± 7

^a^ Data were derived from three independent experiments and express as means ± SD.

## References

[1] Eckert D.M., Kim P.S. (2001). Mechanisms of viral membrane fusion and its inhibition. Annu. Rev. Biochem..

[2] Chan D.C., Kim P.S. (1998). HIV entry and its inhibition. Cell.

[3] Weissenhorn W., Dessen A., Harrison S.C., Skehel J.J., Wiley D.C. (1997). Atomic structure of the ectodomain from HIV-1 gp41. Nature.

[4] Chan D.C., Fass D., Berger J.M., Kim P.S. (1997). Core structure of gp41 from the HIV envelope glycoprotein. Cell.

[5] Qiu Z., Chong H., Yao X., Su Y., Cui S., He Y. (2015). Identification and characterization of a subpocket on the N-trimer of HIV-1 Gp41: Implication for viral entry and drug target. Aids.

[6] Chan D.C., Chutkowski C.T., Kim P.S. (1998). Evidence that a prominent cavity in the coiled coil of HIV type 1 gp41 is an attractive drug target. Proc. Natl. Acad. Sci. USA.

[7] Crespillo S., Camara-Artigas A., Casares S., Morel B., Cobos E.S., Mateo P.L., Mouz N., Martin C.E., Roger M.G., El Habib R. (2014). Single-chain protein mimetics of the N-terminal heptad-repeat region of gp41 with potential as anti-HIV-1 drugs. Proc. Natl. Acad. Sci. USA.

[8] Chu S., Gochin M. (2013). Identification of fragments targeting an alternative pocket on HIV-1 gp41 by NMR screening and similarity searching. Bioorganic Med. Chem. Lett..

[9] He Y. (2013). Synthesized peptide inhibitors of HIV-1 gp41-dependent membrane fusion. Curr. Pharm. Des..

[10] Matthews T., Salgo M., Greenberg M., Chung J., DeMasi R., Bolognesi D. (2004). Enfuvirtide: The first therapy to inhibit the entry of HIV-1 into host CD4 lymphocytes. Nat. Rev. Drug Discov..

[11] Lalezari J.P., Henry K., O’Hearn M., Montaner J.S., Piliero P.J., Trottier B., Walmsley S., Cohen C., Kuritzkes D.R., Eron J.J. (2003). Enfuvirtide, an HIV-1 fusion inhibitor, for drug-resistant HIV infection in North and South America. N. Engl. J. Med..

[12] Rimsky L.T., Shugars D.C., Matthews T.J. (1998). Determinants of human immunodeficiency virus type 1 resistance to gp41-derived inhibitory peptides. J. Virol..

[13] Greenberg M.L., Cammack N. (2004). Resistance to enfuvirtide, the first HIV fusion inhibitor. J. Antimicrob. Chemother..

[14] Wei X., Decker J.M., Liu H., Zhang Z., Arani R.B., Kilby J.M., Saag M.S., Wu X., Shaw G.M., Kappes J.C. (2002). Emergence of resistant human immunodeficiency virus type 1 in patients receiving fusion inhibitor (T-20) monotherapy. Antimicrob. Agents Chemother..

[15] Baldwin C.E., Sanders R.W., Deng Y., Jurriaans S., Lange J.M., Lu M., Berkhout B. (2004). Emergence of a drug-dependent human immunodeficiency virus type 1 variant during therapy with the T20 fusion inhibitor. J. Virol..

[16] Pu J., Wang Q., Xu W., Lu L., Jiang S. (2019). Development of Protein- and Peptide-Based HIV Entry Inhibitors Targeting gp120 or gp41. Viruses.

[17] Otaka A., Nakamura M., Nameki D., Kodama E., Uchiyama S., Nakamura S., Nakano H., Tamamura H., Kobayashi Y., Matsuoka M. (2002). Remodeling of gp41-C34 peptide leads to highly effective inhibitors of the fusion of HIV-1 with target cells. Angew. Chem..

[18] He Y., Xiao Y., Song H., Liang Q., Ju D., Chen X., Lu H., Jing W., Jiang S., Zhang L. (2008). Design and evaluation of sifuvirtide, a novel HIV-1 fusion inhibitor. J. Biol. Chem..

[19] Dwyer J.J., Wilson K.L., Davison D.K., Freel S.A., Seedorff J.E., Wring S.A., Tvermoes N.A., Matthews T.J., Greenberg M.L., Delmedico M.K. (2007). Design of helical, oligomeric HIV-1 fusion inhibitor peptides with potent activity against enfuvirtide-resistant virus. Proc. Natl. Acad. Sci. USA.

[20] Xiong S., Borrego P., Ding X., Zhu Y., Martins A., Chong H., Taveira N., He Y. (2017). A Helical Short-Peptide Fusion Inhibitor with Highly Potent Activity against Human Immunodeficiency Virus Type 1 (HIV-1), HIV-2, and Simian Immunodeficiency Virus. J. Virol..

[21] Chong H., Qiu Z., Su Y., Yang L., He Y. (2015). Design of a highly potent HIV-1 fusion inhibitor targeting the gp41 pocket. Aids.

[22] Chong H., Yao X., Qiu Z., Sun J., Zhang M., Waltersperger S., Wang M., Liu S.L., Cui S., He Y. (2013). Short-peptide fusion inhibitors with high potency against wild-type and enfuvirtide-resistant HIV-1. FASEB J..

[23] Su Y., Chong H., Xiong S., Qiao Y., Qiu Z., He Y. (2015). Genetic Pathway of HIV-1 Resistance to Novel Fusion Inhibitors Targeting the Gp41 Pocket. J. Virol..

[24] Su Y., Chong H., Qiu Z., Xiong S., He Y. (2015). Mechanism of HIV-1 Resistance to Short-Peptide Fusion Inhibitors Targeting the Gp41 Pocket. J. Virol..

[25] Yu D., Ding X., Liu Z., Wu X., Zhu Y., Wei H., Chong H., Cui S., He Y. (2018). Molecular mechanism of HIV-1 resistance to sifuvirtide, a clinical trial-approved membrane fusion inhibitor. J. Biol. Chem..

[26] Wu X., Liu Z., Ding X., Yu D., Wei H., Qin B., Zhu Y., Chong H., Cui S., He Y. (2018). Mechanism of HIV-1 Resistance to an Electronically Constrained alpha-Helical Peptide Membrane Fusion Inhibitor. J. Virol..

[27] Eggink D., Baldwin C.E., Deng Y., Langedijk J.P., Lu M., Sanders R.W., Berkhout B. (2008). Selection of T1249-resistant human immunodeficiency virus type 1 variants. J. Virol..

[28] Eggink D., Bontjer I., Langedijk J.P., Berkhout B., Sanders R.W. (2011). Resistance of human immunodeficiency virus type 1 to a third-generation fusion inhibitor requires multiple mutations in gp41 and is accompanied by a dramatic loss of gp41 function. J. Virol..

[29] Shimura K., Nameki D., Kajiwara K., Watanabe K., Sakagami Y., Oishi S., Fujii N., Matsuoka M., Sarafianos S.G., Kodama E.N. (2010). Resistance profiles of novel electrostatically constrained HIV-1 fusion inhibitors. J. Biol. Chem..

[30] Nameki D., Kodama E., Ikeuchi M., Mabuchi N., Otaka A., Tamamura H., Ohno M., Fujii N., Matsuoka M. (2005). Mutations conferring resistance to human immunodeficiency virus type 1 fusion inhibitors are restricted by gp41 and Rev-responsive element functions. J. Virol..

[31] Geng X., Liu Z., Yu D., Qin B., Zhu Y., Cui S., Chong H., He Y. (2019). Conserved Residue Asn-145 in the C-Terminal Heptad Repeat Region of HIV-1 gp41 is Critical for Viral Fusion and Regulates the Antiviral Activity of Fusion Inhibitors. Viruses.

[32] Zhu Y., Ding X., Yu D., Chong H., He Y. (2019). The Tryptophan-Rich Motif of HIV-1 Gp41 Can Interact with the N-Terminal Deep Pocket Site: New Insights into the Structure and Function of Gp41 and Its Inhibitors. J. Virol..

[33] Lohrengel S., Hermann F., Hagmann I., Oberwinkler H., Scrivano L., Hoffmann C., von Laer D., Dittmar M.T. (2005). Determinants of human immunodeficiency virus type 1 resistance to membrane-anchored gp41-derived peptides. J. Virol..

[34] Sista P.R., Melby T., Davison D., Jin L., Mosier S., Mink M., Nelson E.L., DeMasi R., Cammack N., Salgo M.P. (2004). Characterization of determinants of genotypic and phenotypic resistance to enfuvirtide in baseline and on-treatment HIV-1 isolates. Aids.

[35] Liu Z., Shan M., Li L., Lu L., Meng S., Chen C., He Y., Jiang S., Zhang L. (2011). In vitro selection and characterization of HIV-1 variants with increased resistance to sifuvirtide, a novel HIV-1 fusion inhibitor. J. Biol. Chem..

[36] Cabrera C., Marfil S., Garcia E., Martinez-Picado J., Bonjoch A., Bofill M., Moreno S., Ribera E., Domingo P., Clotet B. (2006). Genetic evolution of gp41 reveals a highly exclusive relationship between codons 36, 38 and 43 in gp41 under long-term enfuvirtide-containing salvage regimen. Aids.

[37] Xu L., Pozniak A., Wildfire A., Stanfield-Oakley S.A., Mosier S.M., Ratcliffe D., Workman J., Joall A., Myers R., Smit E. (2005). Emergence and evolution of enfuvirtide resistance following long-term therapy involves heptad repeat 2 mutations within gp41. Antimicrob. Agents Chemother..

[38] Loutfy M.R., Raboud J.M., Montaner J.S., Antoniou T., Wynhoven B., Smaill F., Rouleau D., Gill J., Schlech W., Brumme Z.L. (2007). Assay of HIV gp41 amino acid sequence to identify baseline variation and mutation development in patients with virologic failure on enfuvirtide. Antivir. Res..

[39] Poveda E., Rodes B., Labernardiere J.L., Benito J.M., Toro C., Gonzalez-Lahoz J., Faudon J.L., Clavel F., Schapiro J., Soriano V. (2004). Evolution of genotypic and phenotypic resistance to Enfuvirtide in HIV-infected patients experiencing prolonged virologic failure. J. Med. Virol..

[40] Ray N., Blackburn L.A., Doms R.W. (2009). HR-2 mutations in human immunodeficiency virus type 1 gp41 restore fusion kinetics delayed by HR-1 mutations that cause clinical resistance to enfuvirtide. J. Virol..

[41] Ray N., Harrison J.E., Blackburn L.A., Martin J.N., Deeks S.G., Doms R.W. (2007). Clinical resistance to enfuvirtide does not affect susceptibility of human immunodeficiency virus type 1 to other classes of entry inhibitors. J. Virol..

[42] Svicher V., Aquaro S., D’Arrigo R., Artese A., Dimonte S., Alcaro S., Santoro M.M., Di Perri G., Caputo S.L., Bellagamba R. (2008). Specific enfuvirtide-associated mutational pathways in HIV-1 Gp41 are significantly correlated with an increase in CD4(+) cell count, despite virological failure. J. Infect. Dis..

[43] Eggink D., Langedijk J.P., Bonvin A.M., Deng Y., Lu M., Berkhout B., Sanders R.W. (2009). Detailed mechanistic insights into HIV-1 sensitivity to three generations of fusion inhibitors. J. Biol. Chem..

[44] Yao X., Chong H., Zhang C., Waltersperger S., Wang M., Cui S., He Y. (2012). Broad antiviral activity and crystal structure of HIV-1 fusion inhibitor sifuvirtide. J. Biol. Chem..

[45] Sivaraman V., Zhang L., Meissner E.G., Jeffrey J.L., Su L. (2009). The heptad repeat 2 domain is a major determinant for enhanced human immunodeficiency virus type 1 (HIV-1) fusion and pathogenicity of a highly pathogenic HIV-1 Env. J. Virol..

[46] Izumi K., Kodama E., Shimura K., Sakagami Y., Watanabe K., Ito S., Watabe T., Terakawa Y., Nishikawa H., Sarafianos S.G. (2009). Design of peptide-based inhibitors for human immunodeficiency virus type 1 strains resistant to T-20. J. Biol. Chem..

[47] Desmezieres E., Gupta N., Vassell R., He Y., Peden K., Sirota L., Yang Z., Wingfield P., Weiss C.D. (2005). Human immunodeficiency virus (HIV) gp41 escape mutants: Cross-resistance to peptide inhibitors of HIV fusion and altered receptor activation of gp120. J. Virol..

[48] De Feo C.J., Wang W., Hsieh M.L., Zhuang M., Vassell R., Weiss C.D. (2014). Resistance to N-peptide fusion inhibitors correlates with thermodynamic stability of the gp41 six-helix bundle but not HIV entry kinetics. Retrovirology.

[49] Chong H., Yao X., Qiu Z., Sun J., Qiao Y., Zhang M., Wang M., Cui S., He Y. (2014). The M-T hook structure increases the potency of HIV-1 fusion inhibitor sifuvirtide and overcomes drug resistance. J. Antimicrob. Chemother..

[50] Chong H., Qiu Z., Sun J., Qiao Y., Li X., He Y. (2014). Two M-T hook residues greatly improve the antiviral activity and resistance profile of the HIV-1 fusion inhibitor SC29EK. Retrovirology.

[51] Wang L.X., Song H., Liu S., Lu H., Jiang S., Ni J., Li H. (2005). Chemoenzymatic synthesis of HIV-1 gp41 glycopeptides: Effects of glycosylation on the anti-HIV activity and alpha-helix bundle-forming ability of peptide C34. Chembiochem.

